# Infective endocarditis in pediatric patients: a decade of insights from a leading Spanish heart surgery reference center

**DOI:** 10.1007/s00431-024-05606-3

**Published:** 2024-06-24

**Authors:** Inés Marín-Cruz, Roberto Pedrero-Tomé, Belén Toral, Marta Flores, María Ángeles Orellana-Miguel, Lorenzo Boni, Sylvia Belda-Hofheinz, Luis M. Prieto-Tato, Elisa Fernández-Cooke, Cristina Epalza, Francisco López-Medrano, Pablo Rojo, Daniel Blázquez-Gamero

**Affiliations:** 1 Pediatric Infectious Diseases, Hospital Universitario 12 de Octubre, Madrid, Spain; 2 Instituto de Investigación Hospital 12 de Octubre (i+12), Madrid, Spain; 3 Pediatric Cardiology, Instituto Pediátrico del Corazón, Hospital Universitario 12 de Octubre, Madrid, Spain; 4 Microbiology, Hospital Universitario 12 de Octubre, Madrid, Spain; 5 Cardiothoracic Surgery, Instituto Pediátrico del Corazón, Hospital Universitario 12 de Octubre, Madrid, Spain; 6 Pediatric Intensive Care Unit, Hospital Universitario 12 de Octubre, Madrid, Spain; 7 Universidad Complutense de Madrid, Madrid, Spain; 8 Mother-Child Health and Development Network (Red SAMID), Carlos-III Health Institute, Madrid, Spain; 9 Unit of Infectious Diseases, Hospital Universitario 12 de Octubre, Madrid, Spain; 10 Centro de Investigación Biomédica en Red de Enfermedades Infecciosas (CIBERINFEC), Madrid, Spain

**Keywords:** Infective endocarditis, Gram-negative bacteria, Multidrug-resistant bacteria, Epidemiology

## Abstract

Infective endocarditis (IE) is a rare disease in children and is associated with significant morbidity and mortality. In recent years, significant changes have occurred in pediatric care that could have influenced the microbiology and presentation of IE. The aim of this work was to study epidemiological, microbiological, and clinical features of IE treated at a Pediatric Cardiac Surgery Reference Center located in Madrid (Spain) in a 10-years’ period. A descriptive observational retrospective study was performed, including pediatric patients < 16 years old with definite or possible IE admitted to a reference center between January 2012 and December 2021. Thirty-two IE episodes were identified. Twenty-eight (87.5%) had congenital heart disease (CHD), 8 (25.0%) were preterm infants, 1 (3.1%) was immunocompromised and 6 (18.8%) had other chronic conditions; in 11 (34.4%) episodes more than one underlying condition was associated. In 20 (62.5%) episodes there was an indwelling central venous catheter (CVC); children with other comorbidities (preterm, immunocompromised, other chronic conditions) were more likely to have a CVC at diagnosis compared with patients with isolated CHD (*p* < 0.001). Thirty-six microbiological isolates were obtained in the 32 episodes; 4 (12.5%) episodes had 2 isolated microorganisms. Microbiological isolates were 20 (55.6%) Gram-positive bacteria (GPB), 10 (27.8%) non-HACEK Gram-negative bacteria (GNB), 1 (2.8%) HACEK-group bacterium, 4 (11.1%) fungi and 1 (2.8%) *Coxiella burnetii*. In 10 (31.3%) episodes, patients were colonized by multidrug-resistant bacteria (MDRB) and the etiology of IE in 3 (30.0%) of those episodes was the colonizing MDRB. MDRB colonization was associated with MDRB IE (*p* = 0.007). The most common complication was septic embolism: 11 (34.4%) episodes (9 pulmonary and 2 cerebral). In-hospital mortality was 6.3% (*n* = 2), all of them due to underlying conditions and not to IE or its complications. Clinical features and complications of IE episodes caused by non-HACEK GNB and those caused by GPB were compared, finding no statistically significant differences.

*    Conclusion*: Risk factors for developing IE, the proportion of embolic complications, and mortality rate were consistent with previously published findings. Proportion of IE cases attributed to non-HACEK GNB was higher than previously reported, suggesting an evolving epidemiology of IE. One-third of children colonized with MDRB subsequently developed IE caused by the same MDRB strains, so empirical coverage of MDRB organisms must be considered when IE is suspected in MDRB colonized patients. No significant differences in clinical features and complications were observed when comparing IE episodes caused by non-HACEK GNB and those caused by GPB, however larger cohort studies are needed.

**Table:** 

**What is Known:** *• Infective endocarditis (IE) is a rare disease in children, associated with significant morbidity and mortality.* *• The main risk factor for developing IE in children is an underlying congenital heart disease.*	
**What is New:** *• With current changing epidemiology in pediatric IE, a higher proportion of IE caused by non-HACEK Gram-negative bacteria should be expected.* *• A significant percentage of children colonized by multidrug-resistant bacteria can develop an IE due to those bacteria.*

**What is Known:**

*• Infective endocarditis (IE) is a rare disease in children, associated with significant morbidity and mortality.*

*• The main risk factor for developing IE in children is an underlying congenital heart disease.*

**What is New:**

*• With current changing epidemiology in pediatric IE, a higher proportion of IE caused by non-HACEK Gram-negative bacteria should be expected.*

*• A significant percentage of children colonized by multidrug-resistant bacteria can develop an IE due to those bacteria.*

## Introduction

Infective endocarditis (IE) is an infection of the cardiac endothelium that has low incidence in the pediatric population; however, it has an important morbidity and mortality (described mortality of 5–10% [1, 2]).

Congenital heart disease (CHD) is currently the main risk factor to develop IE in children (as much as 15–140 times higher compared to general population [3, 4]), due to the increase in survival in these patients that currently undergo surgical corrections that previously were not technically possible [5, 6] and the presence of prosthetic material after corrective surgery [7, 8]. However, with the increasing complexity in healthcare in pediatrics, a rise of IE in pediatric patients without CHD has been described [2], being other risk factors associated with pediatric IE prolonged indwelling central venous catheter (CVC) [9–11], immunocompromised patients (especially those receiving oncological treatment [12] or hematopoietic stem cell transplantation (HSCT) recipients [13]) or pediatric patients admitted to neonatal or pediatric intensive care units (NICU, PICU) that undergo invasive procedures [14, 15].

*Staphylococcus aureus* and viridans streptococci are still the leading causes of pediatric IE [2, 4, 5], nonetheless in recent years there has been an increase in pediatric IE caused by other less frequent microorganisms, such as coagulase-negative staphylococci (CoNS), Gram-negative bacteria (GNB), and fungi [11, 16]. Risk factors for CoNS IE are prosthetic material and prolonged indwelling CVC [5]. Neonatal period and prolonged indwelling CVC are risk factors for GNB IE [5]. NICU admission (especially in preterm infants), cancer patients, and prolonged indwelling CVC are well described risk factors for fungal IE [17, 18].

In pediatric IE, the complication with the highest morbidity is septic embolism, which can occur up to 40% of the cases (higher risk than adult population) [19]. The reported in-hospital mortality in pediatric series of IE ranges from 5 to 10%; with higher rates in patients with *S. aureus* IE, preterm infants (31%), patients with tetralogy of Fallot and pulmonary atresia (48%) and patients with prosthetic valve IE (8%) [1, 2, 10, 20].

It is important to have an updated knowledge of IE epidemiology, risk factors, clinical features, and complications in children in order to provide an adequate management. The aim of the present study is to describe these features of the IE episodes treated at a tertiary Spanish pediatric hospital in a 10-years’ period.

## Materials and methods

### Study design

An observational retrospective study was performed, describing the characteristics of pediatric patients (< 16 years old) with IE treated at a tertiary hospital in Madrid (Spain), national reference for complex CHD patients with advanced heart surgery, between January 2012 and December 2021.

### Patients

Patients were eligible if they fulfilled all the following inclusion criteria: age < 16 years, treated at any unit at Hospital Universitario 12 de Octubre, with definite or possible IE (according to the ESC 2015 modified Duke criteria [21]).

### Clinical data

Clinical data were obtained from the review of informatized clinical records and collected in a REDCap online database elaborated for this study. Patients’ date of birth, gender, date of diagnosis, fulfilled modified Duke criteria, and underlying conditions were noted. Also, previous surgical correction of CHD, presence of indwelling CVC, and multidrug-resistant bacteria (MDRB) colonization were recorded. Recorded data of the IE episodes included clinical presentation, echocardiographic findings, and microbiologic data. Also, medical, and surgical treatment, complications, length of hospitalization, and outcomes were noted.

### Statistical analysis

Categorical variables were reported as frequencies and percentages, and continuous variables as median and interquartile range (IQR). To evaluate the possible association between microorganism and clinical presentation and complications, Fisher’s exact test and Wilcoxon-Mann-Whitney test were used. The significance level was α = 0.05. Statistical analysis was performed using IBM SPSS Statistics 25.

## Results

### Demographic features

During the study period, 32 episodes of IE were identified (demographic and clinical features shown in Table 1); considering the Duke modified criteria, 28 (87.5%) were definite IE and 4 (12.5%) possible IE.

**Table 1:** Demographic and clinical features of the 32 episodes of infective endocarditis (IE)

	**IE episodes** **(*n* = 32)**
**Gender (male), *n* (%)**	17 (53.1)
**Age, median (IQR), months**	9.1 (4.2–129.8)
**Underlying condition**	
Congenital heart disease, *n* (%)	28 (87.5)
Prematurity, *n* (%)	8 (25.0)
Immunocompromised, *n* (%)	1 (3.1)
Other chronic conditions, *n* (%)	6 (18.8)
> 1 underlying condition, *n* (%)	11 (34.4)
**Central venous catheter, *n* (%)**	20 (62.5)
**Presenting signs**	
Fever, n (%)	27 (84.4)
Shock, n (%)	16 (50.0)
Heart failure, n (%)	1 (3.1)
Vascular phenomena, n (%)	14 (43.8)
Immunological phenomena, n (%)	1 (3.1)

The median age at diagnosis was 9.1 (4.2–129.8) months; 17 (53.1%) episodes were diagnosed in infants younger than 1 year of age, 3 (9.4%) in patients between 1–5 years, 4 (12.5%) in patients between 6–10 years and 8 (25.0%) in patients between 11–15 years.

Regarding underlying conditions, 28 (87.5%) patients had CHD (Table 2), 8 (25.0%) were preterm infants (median gestational age at birth: 27.5 weeks, IQR: 25.0–32.8 weeks), 1 (3.1%) was immunocompromised (metastatic medulloblastoma) and 6 (18.8%) had other chronic conditions (3 had a polymalformative syndrome, 1 short bowel syndrome, 1 esophageal atresia, and 1 Down syndrome); in 11 (34.4%) episodes, more than one underlying condition was associated (6 episodes: CHD plus preterm birth, 5 episodes: CHD plus other chronic conditions).

**Table 2:** Congenital heart disease (CHD) diagnosis

	**CHD (*n* = 28)**
**Cyanotic heart defects, *n* (%)**	**14 (50.0)**
Pulmonary atresia, *n* (%)	5 (17.9)
Hypoplastic left heart syndrome, *n* (%)	4 (14.3)
Double outlet right ventricle, *n* (%)	3 (10.7)
Tetralogy of Fallot, *n* (%)	1 (3.6)
Truncus arteriosus, n (%)	1 (3.6)
**Acyanotic heart defects, *n* (%)**	**14 (50.0)**
Patent ductus arteriosus, *n* (%)	3 (10.7)
Ventricular septal defect, *n* (%)	3 (10.7)
Atrial septal defect, *n* (%)	2 (7.1)
Aortic stenosis, *n* (%)	2 (7.1)
Bicuspid aortic valve, *n* (%)	2 (7.1)
Atrioventricular canal, *n* (%)	1 (3.6)
Persistent valve of coronary sinus, *n* (%)	1 (3.6)

Regarding the 28 episodes of IE diagnosed in patients with CHD, a corrective cardiac surgery or therapeutic cardiac catheterization was previously performed in 20 (71.4%). The median time of IE diagnosis after the cardiac procedure was 47.5 (18.3–348.0) days. At diagnosis, in 20 (62.5%) episodes the patients had an indwelling CVC, and the median time of IE diagnosis after CVC placement was 8.5 (4.0–14.3) days. Eight out of 8 (100%) preterm infants, 1 out of 1 (100%) immunocompromised patient and 5 out of 6 (83.3%) patients with other chronic conditions had an indwelling CVC at diagnosis (i.e., 14 out of 15, 93.3%), compared to 6 out of 17 (35.3%) patients with isolated CHD that had and indwelling CVC at diagnosis (*p* < 0.001).

### Clinical manifestations

Presenting signs were fever in 27 (84.4%) episodes, shock in 16 (50.0%), heart failure in 1 (3.1%), vascular phenomena in 14 (43.8%) (11 (34.4%) septic embolisms, 2 (6.3%) intracranial hemorrhage, 1 (3.1%) Janeway’s lesions), immunological phenomena in 1 (3.1%) (glomerulonephritis and rheumatoid factor elevation in the same episode). The presenting signs in preterm infants compared to other patients were different: fever was only present in 4 out of 8 (50.0%) compared to 23 out of 24 (95.8%) of the non-preterm patients (*p* < 0.001) and shock was present in 7 out of 8 (87.5%) compared to 9 out of 24 (37.5%) of the non-preterm patients (*p* < 0.001).

### Echocardiogram

An echocardiogram was performed in all episodes, transthoracic echocardiography was used in 29 (90.6%) episodes and both transthoracic and transesophageal echocardiography was used in 3 (9.4%). There were echocardiographic findings in 27 (84.4%), which are shown in Table 3.

**Table 3:** Echocardiographic findings

	**Performed echocardiograms (*n* = 32)**
**Right IE, *n* (%)**	**21 (65.6)**
Native tricuspid valve, *n* (%)	10 (31.3)
Entrance of the venae cavae, *n* (%)	3 (9.4)
Prosthetic pulmonary valve, *n* (%)	3 (9.4)
Prosthetic pulmonary conduit, *n* (%)	2 (6.3)
Interatrial septum (right atrium), *n* (%)	1 (3.1)
Interventricular septum (right ventricle), *n* (%)	1 (3.1)
Right chambers, *n* (%)	1 (3.1)
**Left IE, *n* (%)**	**6 (18.8)**
Native aortic valve, *n* (%)	3 (9.4)
Interventricular septum (left ventricle), *n* (%)	1 (3.1)
Ventricular septal defect, *n* (%)	1 (3.1)
Origin of left carotid artery, *n* (%)	1 (3.1)
**Negative echocardiogram, *n* (%)**	**5 (15.6)**

### Microbiology

Thirty-six microbiological isolates were obtained in the 32 episodes (Table 4).

**Table 4:** Isolated microorganisms of the 32 episodes of infective endocarditis

	**Isolated microorganisms** **(*n* = 36**^a^**)**
**GRAM-POSITIVE BACTERIA, *N* (%)**	**20 (55.6)**
Coagulase-negative staphylococci, *n* (%)	7 (19.4)
*Staphylococcus aureus*, *n* (%)	6 (16.7)
*Enterococcus faecalis*, *n* (%)	4 (11.1)
Viridans streptococci, *n* (%)	3 (8.3)
**GRAM-NEGATIVE BACTERIA, *N* (%)**	**11 (30.6)**
** Non-HACEK, *n* (%)**	**10 (27.8)**
Non-fermenters, *n* (%)	5 (13.9)
*Pseudomonas aeruginosa*, *n* (%)	4 (11.1)
*Stenotrophomonas maltophilia*, *n* (%)	1 (2.8)
Enterobacteriaceae, *n* (%)	5 (13.9)
*Escherichia coli*, *n* (%)	2 (5.6)
*Klebsiella pneumoniae*, *n* (%)	1 (2.8)
*Enterobacter cloacae*, *n* (%)	1 (2.8)
*Serratia marcescens*, *n* (%)	1 (2.8)
** HACEK, *n* (%)**	**1 (2.8)**
*Aggregatibacter aphrophilus*, *n* (%)	1 (2.8)
**FUNGI, *N* (%)**	**4 (11.1)**
*Candida albicans*, *n* (%)	2 (5.6)
*Trichosporon inkin*, *n* (%)	2 (5.6)
***COXIELLA BURNETII*, *N* (%)**	**1 (2.8)**

^a^Four episodes with 2 isolates

Blood cultures were positive in 30 (93.8%) episodes; in 4 (12.5%) episodes, more than one microorganism was isolated (2 microorganisms isolated per episode). In those 4 episodes, the isolated microorganisms were: *Staphylococcus aureus* and *Enterococcus faecalis*, *Enterococcus faecalis* and *Staphylococcus epidermidis*, *Enterococcus faecalis *and extended spectrum beta-lactamase (ESBL) *Escherichia coli*, and *Stenotrophomonas maltophilia* and carbapenemase-producing *Klebsiella pneumoniae*. In those 2 episodes without positive blood cultures, *Trichosporon inkin* was isolated at surgical specimen in one and serology for *Coxiella burnetii* was positive (confirmed by PCR in surgical specimen and blood) in another case.

All *S. aureus* isolates were methicillin-sensitive and all *E. faecalis* isolates were ampicillin-sensitive. Among those 10 non-HACEK GNB, 5 (13.9%) were MDRB: 1 MDR *Pseudomonas aeruginosa*, 1 ESBL *Escherichia coli*, 1 carbapenemase-producing *Klebsiella pneumoniae*, and 2 AmpC beta-lactamase producing GNB (*Enterobacter cloacae* and *Serratia marcescens*).

In 10 (31.3%) episodes, patients were previously colonized by MDRB (clinical features of those patients and comparison with non-colonized patients in Table 5). Of those 10 patients, the colonizing bacteria were isolated in 3 (30.0%) of the patients’ blood cultures: 1 MDR *Pseudomonas aeruginosa*, 1 ESBL *Escherichia coli*, and 1 AmpC beta-lactamase producing *Enterobacter cloacae*. MDRB colonization was associated with MDRB IE (p = 0.007), with the presence of indwelling CVC (p = 0.030), and with longer time of PICU/NICU stay (p = 0.005).

**Table 5:** Clinical features of multidrug-resistant bacteria (MDRB) colonized patients and comparison with non-colonized patients

	**MDRB colonized patients (*n* = 10)**	**Non-colonized patients (*n* = 22)**	***p***
**Gender (male), *n* (%)**	3 (30.0)	14 (63.6)	0.077
**Age, median (IQR), months**	6.5 (5.0–28.1)	43.9 (1.7–157.2)	0.393
**Underlying condition**			
Congenital heart disease, *n* (%)	9 (90.0)	19 (86.4)	0.773
Prematurity, *n* (%)	2 (25.0)	6 (27.3)	0.660
Immunocompromised, *n* (%)	1 (10.0)	0 (0)	0.132
Other chronic conditions, *n* (%)	2 (20.0)	4 (18.2)	0.903
> 1 underlying condition, *n* (%)	4 (40.0)	7 (31.8)	0.652
**Central venous catheter, *n* (%)**	9 (90.0)	11 (50.0)	0.030*
Time after CVC placement, median (IQR), days	4.0 (1.5–15.0)	9.0 (6.0–15.0)	0.334
**Intensive care unit admission, *n* (%)**	9 (90.0)	17 (77.3)	0.393
Time of PICU/NICU stay, median (IQR), days	171.0 (57.0–233.0)	23.0 (4.5–58.5)	0.005*
**MDRB IE, *n* (%)**	3 (30.0)	0 (0)	0.007*

Regarding the 4 episodes of fungal IE, one *Candida albicans* IE occurred in a premature newborn (25 weeks of gestational age at birth) with a patent ductus arteriosus and a 20-days’ CVC, the other in an infant with esophageal atresia and an 8-days’ CVC, and both *Trichosporon inkin* IE occurred in an adolescent with pulmonary atresia without CVC but with a prosthetic pulmonary conduit (relapse). All of them where right IE, located at native tricuspid valve, interventricular septum (right ventricle), and prosthetic pulmonary conduit respectively. Both patients with *C. albicans* IE where previously admitted to NICU/PICU. Both the patient with *Trichosporon inkin* IE and the infant with esophageal atresia and *C. albicans* IE had pulmonary embolisms and both required surgical treatment.

### Treatment

The median of total antibiotic treatment duration was 47.0 (IQR 34.8–55.5) days, with a median duration of empirical antibiotic treatment of 4.5 (IQR 3.0–12.0) days. The median delay in initiating antibiotic treatment after the symptoms’ onset was 2.0 (IQR 0.0–5.0) days. The most used empirical antibiotic regimen was meropenem plus a glycopeptide (vancomycin or teicoplanin) with or without an aminoglycoside (gentamicin or amikacin) in 6 (18.8%) episodes, followed by a glycopeptide (vancomycin or teicoplanin) plus an aminoglycoside (gentamicin or amikacin) in 5 (15.6%).

Surgical management was required in 11 (34.4%) episodes, with a median time from diagnosis to surgical intervention of 5.0 (IQR 2.0–22.0) days. The indication of surgery was the difficult to eradicate isolated microorganism in 6 (18.8%) episodes (3 (9.4%) *S. aureus*, 2 (6.3%) *P. aeruginosa*, 1 (3.1%) *C. albicans*; with persistent bacteriemia in 5 (15.6%)), embolism prevention in 2 (6.3%), severe valve regurgitation in 2 (6.3%), and severe pulmonary conduit obstruction in 1 (3.1%).

### Complications

PICU/NICU admission related to IE or its complications was required in 26 (81.3%) episodes, with a median stay of 26.0 (6.5–87.3) days. Complications secondary to IE were present in 25 (78.1%) episodes, 17 (53.1%) of the episodes with more than one complication. Registered complications were septic embolisms in 11 (34.4%) (8 (25.0%) pulmonary, 1 (3.1%) pulmonary and soft tissue, 1 (3.1%) cerebral, 1 (3.1%) cerebral and hepatic), shock in 8 (25.0%), heart failure in 7 (21.9%), valve regurgitation in 7 (21.9%), acute renal failure in 4 (12.5%), neurological complications in 3 (9.4%), local extension of infection in 3 (9.4%), and heart rhythm disturbances in 1 (3.1%). In-hospital mortality occurred in 2 (6.3%) episodes which was not attributed to IE or its complications, but to complications related to the patients’ underlying condition (pulmonary atresia and polymalformative syndrome (CHARGE syndrome) respectively).

### Outcomes

There were 6 (18.8%) recurrences: 4 (12.5%) in-hospital relapses and 2 (6.3%) outpatient recurrences (1 relapse, 1 reinfection). At discharge, there were sequelae in 10 (31.3%) episodes, 3 (9.4%) of the episodes with more than one sequela: valvular regurgitation in 7 (21.9%) (2 (6.3%) of them associating heart failure), neurological sequelae in 3 (9.4%), heart rhythm disturbances in 1 (3.1%), valvular double lesion in 1 (3.1%), and chronic renal failure in 1 (3.1%). At follow-up (median time 3.4 years, IQR 0.7–7.2 years), 6 (18.8%) of those patients had persistent sequelae (1 (3.1%) with more than one sequela): valvular regurgitation in 4 (12.5%), heart failure in 2 (6.3%), neurological sequelae in 1 (3.1%), heart rhythm disturbances in 1 (3.1%), and chronic renal failure in 1 (3.1%).

### Comparison of clinical features and complications depending on causative microorganism of IE

Clinical features and complications of IE episodes caused by non-HACEK GNB were compared to those episodes caused by GPB, and no statistically significant differences were found between them (Table 6).

**Table 6:** Comparison of clinical features and complications depending on causative microorganism of IE

	**Bacterial IE**^a^ **(*n* = 26)**	**Gram-positive bacteria (*n* = 17**^b^**)**	**Gram-negative bacteria (*n* = 9**^c^**)**	***p***
**Gender (male)**	14 (53.8)	9 (52.9)	5 (55.6)	0.899
**Age, median (IQR), months**	6.5 (3.5–67.9)	18.0 (2.8–153.4)	5.9 (3.5–37.4)	0.467
**Underlying condition**				
Congenital heart disease, *n* (%)	23 (88.5)	15 (88.2)	8 (88.9)	0.960
Prematurity, *n* (%)	7 (26.9)	4 (23.5)	3 (33.3)	0.592
Immunocompromised, *n* (%)	1 (3.8)	1 (5.9)	0 (0.0)	0.458
Other chronic conditions, *n* (%)	5 (19.2)	2 (11.8)	3 (33.3)	0.184
> 1 underlying condition, *n* (%)	10 (38.5)	5 (29.4)	5 (55.6)	0.192
**Central venous catheter, *n* (%)**	18 (69.2)	10 (58.8)	8 (88.9)	0.114
**Presenting signs**				
Fever, *n* (%)	22 (84.6)	14 (82.4)	8 (88.9)	0.660
Shock, *n* (%)	15 (57.7)	8 (47.1)	7 (77.8)	0.131
Vascular phenomena, *n* (%)	9 (34.6)	6 (35.3)	3 (33.3)	0.920
Immunological phenomena, *n* (%)	1 (3.8)	1 (5.9)	0 (0.0)	0.458
**Echocardiogram**				
Positive echocardiogram, *n* (%)	22 (84.6)	16 (94.1)	6 (66.7)	0.065
Left IE, *n* (%)	5 (19.2)	4 (23.5)	1 (11.1)	0.169
Right IE, *n* (%)	17 (65.4)	12 (70.6)	5 (55.6)	0.169
**Complications**				
PICU admission, *n* (%)	21 (80.8)	12 (70.6)	9 (100)	0.070
Surgical management, *n* (%)	7 (26.9)	5 (29.4)	2 (22.2)	0.694
Septic embolisms, *n* (%)	7 (26.9)	4 (23.5)	3 (33.3)	0.592
Local extension of infection, *n* (%)	1 (3.8)	1 (5.9)	0 (0.0)	0.458
Heart failure, *n* (%)	5 (19.2)	2 (11.8)	3 (33.3)	0.184
Residual valve regurgitation, *n* (%)	5 (19.2)	5 (29.4)	0 (0.0)	0.070
Heart rhythm disturbances, *n* (%)	1 (3.8)	1 (5.9)	0 (0.0)	0.458
Neurological complications, *n* (%)	3 (11.5)	1 (5.9)	2 (22.2)	0.215
Acute renal failure, *n* (%)	3 (11.5)	3 (17.6)	0 (0.0)	0.180
Recurrences, *n* (%)	4 (15.4)	2 (11.8)	2 (22.2)	0.482
Mortality, *n* (%)	2 (7.7)	1 (5.9)	1 (11.1)	0.634

^a^Excluding *Coxiella burnetii* IE and *Aggregatibacter aphrophilus* IE

^b^The two episodes with two Gram-positive bacteria isolates were counted as one Gram-positive bacteria IE each

^c^The episode with two Gram-negative bacteria isolates and the episode with one Gram-negative bacteria and one Gram-positive bacteria isolates were counted as one Gram-negative bacteria IE each

Although children with non-HACEK GNB IE exhibited a higher proportion of PICU admission (100% vs. 70.6%), shock (77.8% vs. 47.1%), heart failure (33.3% vs. 11.8%), neurological complications (22.2% vs. 5.9%), more than one underlying condition (55.6% vs. 29.4%), and presence of indwelling CVC (88.9% vs. 58.8%), these distinctions did not reach statistical significance. On the other hand, children with GPB IE showed a trend towards higher rate of residual valve regurgitation (29.4% vs 0.0%).

## Discussion

Although IE is a rare disease in childhood, its incidence is increasing in the last years due to increase in health-care complexity [2]. For this same reason, epidemiology, risk factors and causative microorganisms are changing [11], so it is of paramount importance to have an updated knowledge of them in our setting to manage this disease adequately. We designed our study to describe the characteristics of the pediatric IE episodes treated in a cardiology reference center in a 10 years’ period.

As shown in previous reports [5], the main underlying condition was CHD (87.5%), suggesting that CHD is the main risk factor to develop IE in the pediatric population. The second risk factor for developing IE (25.0%), was being preterm (especially those extremely preterm, with a median of 27.5 weeks of gestational age at birth in our study); which has been also previously published as risk factor due to the prolonged indwelling CVC [9] and the fact that they undergo invasive procedures [14, 15]. In our study, only 3.1% episodes occurred in immunocompromised children, a population that may have been underrepresented due to the characteristics of our center, where HSCT (a well described risk factor for developing IE in children [13]) is not performed. Additionally, indwelling CVC was present in 93.3% of the recorded IE episodes in patients that were preterm, immunocompromised or had other chronic conditions compared to 35.3% episodes where CHD was the only underlying condition. This finding is in line with other studies where indwelling CVC is the main risk factor to develop an IE in these 3 groups of patients [9–11]. In our series, there were two incidence peaks regarding age at presentation: 53.1% presented in patients younger than 1 year and 25.0% in patients older than 10 years, similar to what is reported in previous literature where episodes peak in the first year of life (where surgical correction of the more severe CHD is performed) and adolescence [2].

Regarding clinical manifestations, we found that the preterm infant group had significantly different clinical presentation compared to the other patients: only 50.0% of preterm infants in our study had fever (vs. 95.8% in non-preterm children) and 87.5% presented with shock (vs. 37.5% in non-preterm children). These findings suggest that maybe other diagnostic criteria of IE should be applied in preterm patients, as previously has been proposed by other group [22].

Regarding microbiological isolates, the most frequent isolates were CoNS (19.4%) and *S. aureus* (16.7%) in contrast with what is reported in the literature [2, 4, 5, 20] and other recent series [23, 24] where the most frequently isolated microorganisms are *S. aureus* and viridans streptococci (which in our series were only isolated in 8.3% of the episodes). Remarkably, our study showed an important proportion of non-HACEK GNB (27.8%) which is significantly above what has been previously reported (0–9.1%) [23, 25, 26]. The proportion of fungal IE in our series (11.1%) was similar to other Spanish series [25, 27], being *C. albicans* the most frequent isolate [5].

Of note, we registered the MDRB colonization. Among 10 MDRB colonized children with IE, 3 (30.0%) had the same bacteria in blood cultures. This significantly high proportion of MDRB IE has not been previously reported in pediatric IE literature. Due to this finding, it might be advisable to consider empirical antibiotic coverage of these MDRB when suspecting IE in colonized patients. MDRB colonization was also associated with the presence of CVC and with longer NICU/PICU stay. Children with severe CHD spend long periods admitted to NICU/PICU and those with chronic conditions need a prolonged indwelling CVC, both situations with high risk for MDRB colonization and therefore possibility for MDRB IE.

Regarding IE complications, the main complication was septic embolism, present in 34.4% (*n* = 11) of the episodes (28.1% pulmonary embolisms and 6.3% cerebral embolisms). Proportion of embolism was similar to that reported in other pediatric study (40%) [18], although in our study the proportion of cerebral embolisms was lower (6.3% vs 20%) and that of pulmonary embolisms was higher (28.1% vs 8%), probably due to a lower proportion of left IE in our cohort (18.8% vs 55.9%). Previous studies showed that left sided IE have a higher risk for cerebral embolism compared to right IE (32% vs 2.8%) [28].

In-hospital mortality in our study was 6.3% (*n* = 2), like that reported in other studies [1, 2]. None of the deaths was due to IE or its complications. One of the patients had pulmonary atresia (a described risk factor for higher mortality -48%- [2]) and the other patient had a polymalformative syndrome. In our study, 18.8% (*n* = 6) of the patients had persistent sequelae at follow-up. Mortality and sequelae rates are consistent with previous literature [1, 8, 11, 23–25]. Despite IE is a rare disease, long-term sequelae in children with IE are still common, which may be relevant in populations with higher risk of IE, like patients with CHD or preterm infants.

We did not find statistically significant differences in clinical features or in complication rates between non-HACEK GNB and GPB IE, probably due to a limited sample size, but we could observe some interesting trends in this study (Table 6). There was a higher proportion of non-HACEK GNB IE in children with more than one underlying condition (55.6%) and children with indwelling CVC (88.9%), probably due to frequent contact with the health-care system. Non-HACEK GNB IE showed a higher proportion of PICU admission (100%), shock (77.8%), heart failure (33.3%) and neurologic complications (22.2%), which may be due to their higher virulence. On the other hand, GPB IE showed a higher proportion of residual valve regurgitation (29.4%), which may be caused by tendency of biofilm formation in GPB with higher potential for valve disruption.

Limitations of our study are those inherent to retrospective studies (missing data, prone to biases). Limited sample size is a major limitation of the study as IE is a rare condition in children, even in a reference center with advanced heart surgery like in this study.

In conclusion, risk factors for developing IE, the proportion of embolic complications, and mortality rate were consistent with previously published findings. Notably, the proportion of IE cases attributed to non-HACEK GNB was higher than previously reported, suggesting an evolving epidemiology of IE. It is noteworthy that one-third of children colonized with MDRB subsequently developed IE caused by the same MDRB strains; this emphasizes the potential importance of considering empiric antibiotic coverage against colonizing MDRB in those patients. While no statistically significant differences in clinical features and complications were observed when comparing IE episodes caused by non-HACEK GNB and those caused by GPB, larger cohort studies are needed for further investigation.

## Data Availability

No datasets were generated or analysed during the current study.

## Abbreviations

CHD: Congenital heart disease
CoNS: Coagulase-negative staphylococci
CVC: Central venous catheter
ESBL: Extended spectrum beta-lactamase
GNB: Gram-negative bacteria
GPB: Gram-positive bacteria
HSCT: Hematopoietic stem cell transplantation
IE: Infective endocarditis
IQR: Interquartile range
MDRB: Multidrug-resistant bacteria
NICU: Neonatal intensive care unit
PICU: Pediatric intensive care unit
